# Treatment of an Adolescent Female With Nonalcoholic Steatohepatitis–Related Cirrhosis With Liraglutide

**DOI:** 10.1097/PG9.0000000000000303

**Published:** 2023-03-30

**Authors:** Christina Yuen, Tiffany Yu, Samuel French, Elizabeth A. Marcus, Joanna Yeh, Harvey Chiu

**Affiliations:** From the * Department of Pediatrics, University of California, Los Angeles Mattel Children’s Hospital, Los Angeles, CA; † Department of Radiological Sciences, Ronald Reagan University of California Los Angeles Medical Center, Los Angeles, CA; ‡ Department of Pathology and Laboratory Medicine, Ronald Reagan University of California Los Angeles Medical Center, Los Angeles, CA; § Department of Pediatric Gastroenterology, University of California, Los Angeles Mattel Children’s Hospital, Los Angeles, CA; ∥ Department of Pediatric Endocrinology, University of California, Los Angeles Mattel Children’s Hospital, Los Angeles, CA.

**Keywords:** obesity, weight loss, liver fibrosis

## Abstract

Nonalcoholic fatty liver disease is the most common chronic liver disease in children in the United States and encompasses a range of disease from steatosis to cirrhosis. The mainstay of treatment is lifestyle modifications like increased physical activity and healthier eating habits. These are sometimes augmented with medications or surgery for weight loss. We present a patient with biopsy-proven nonalcoholic steatohepatitis-related cirrhosis that did not improve with suboptimal lifestyle changes. This patient’s disease progression reversed after liraglutide treatment, as evidenced by improved imaging and laboratory results, despite no significant improvement in her body mass index percentile. This case demonstrates the importance of considering liraglutide for patients with nonalcoholic steatohepatitis and suggests a hepatic effect independent of effects related to weight loss.

## INTRODUCTION

Nonalcoholic fatty liver disease (NAFLD) is the most common chronic liver disease in children in the United States (1). NAFLD refers to a spectrum, from simple steatosis characterized by liver fat accumulation to nonalcoholic steatohepatitis (NASH) characterized by elevated transaminase levels to cirrhosis. This progression can be reflected by an increased liver fat fraction on magnetic resonance imaging (MRI) and increased liver stiffness on elastography (2).

Treatment for NAFLD centers on lifestyle modifications, including increased physical activity and healthy eating habits. For patients where these are not sufficient, interventions that induce weight loss like bariatric surgery or medications can be considered (1). Liraglutide, a glucagon-like peptide-1 receptor agonist, was approved by the Food and Drug Administration for obesity treatment in adolescents aged 12 years or older. We report a patient with biopsy-proven NASH-related cirrhosis without improvement after lifestyle interventions who had decreased transaminase levels and improved NASH findings on MRI without significant weight loss after liraglutide treatment.

## CASE PRESENTATION

A 9-year-old female with obesity (body mass index [BMI] 98th percentile) and pre-diabetes mellitus (hemoglobin A1c 5.7%) presented to pediatric gastroenterology clinic due to an elevated alanine transaminase (ALT) of 120 U/L (Table 1).

**TABLE 1.** Comparison of the patient’s hemoglobin A1c, liver enzymes, BMI, BMI percentile, and BMI *Z* score at specified patient events

	Initial presentation to GI clinic (time = 0)	Initial liver biopsy (time = +5 mo)	Repeat liver biopsy (time = +25 mo)	MRI with evidence of cirrhosis (time = +27 mo)	Before starting liraglutide (time = +39 mo)	After liraglutide 1.8 mg/d (time = +43 mo)	After liraglutide 2.4–3 mg/d (time = +47 mo)	After 2 y of liraglutide (time = +62 mo)	Normal ranges
Hgb A1c, %	5.7	5.8	6.1	6.1	6.3	6.1	6.1	5.4	<5.7
AST, U/L	82	71	53	54	99	56	48	28	<34
ALT, U/L	120	104	74	71	129	71	57	28	<20
BMI, kg/m^2^	25.02	26.2	27.87	28.16	29.2	27.88	27.73	29.65	
BMI percentile, %	97.97	98.22	97.88	97.94	97.78	96.66	96.35	96.9	
BMI *Z* score	2.05	2.10	2.03	2.04	2.01	1.83	1.79	1.87	

ALT = alanine transaminase; AST = aspartate transaminase; BMI = body mass index; GI = gastroenterology; Hgb A1c = hemoglobin A1c; MRI = magnetic resonance imaging.

**FIGURE 1.**
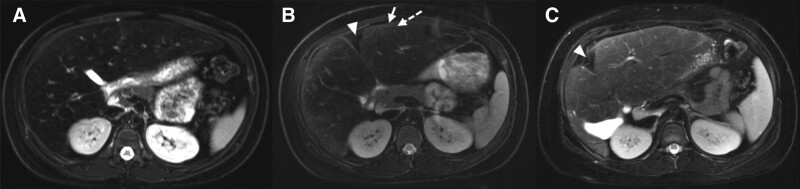
MRI abdomen T2 fat saturation axial sequences from the patient’s (A) initial MRI, (B) second MRI, and (C) most recent MRI demonstrate cirrhosis, with notable fibrosis progression from the initial to second MRI, and grossly stable imaging findings from the second MRI to the most recent MRI. The presence of subtle nodular hepatic contour (arrow), widening of the intrahepatic fissure (arrowhead), and subtle linear T2 hyperintense parenchymal bands represents areas of fibrosis (dashed arrow). Decreased fat infiltration in (C) is evidenced by the brighter signal in the liver. MRI = magnetic resonance imaging.

**FIGURE 2.**
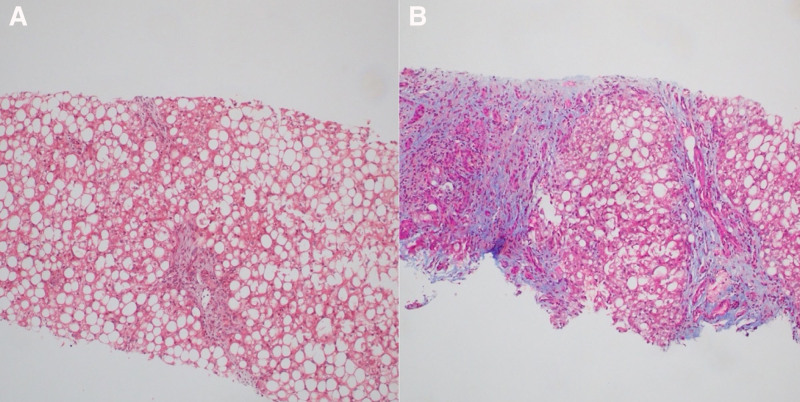
(A) The patient’s initial liver biopsy shows increased steatosis and periportal fibrosis (stage 1) (trichome staining), and (B) repeat liver biopsy shows progression of disease, evidenced by steatohepatitis with extensive bridging fibrosis, suggesting early compensated cirrhosis (trichome staining). Staining of both biopsies showed no alpha-1 antitrypsin globules or pathologic iron accumulation.

Abdominal MRI at this time showed hepatomegaly (Fig. 1A). Liver fat fraction obtained via multi-echo Dixon VIBE sequences was greater than 30% (normal <5.6%). Liver stiffness was 2.5 kPa (2.5–2.9 kPa may be normal or represent inflammation). Given her slightly elevated hemoglobin A1c (Hgb A1c) that peaked at 6.4%, metformin was started off-label for weight loss to augment lifestyle changes. Her initial liver biopsy 5 months after presentation showed steatohepatitis with periportal fibrosis (stage 1) (Fig. 2A). Further laboratory evaluation to rule out other causes of hepatitis was negative.

Despite lifestyle modifications and metformin use over 1 year, the patient’s BMI percentile and ALT remained elevated at the 98th percentile and 104 U/L, respectively. An MRI 19 months after presentation showed a stable liver fat fraction of 29.3% and worsened liver stiffness of 3.8–4.6 kPa (3.5–4 kPa correlates to stage 2–3 fibrosis, 4–5 kPa correlates to stage 3–4 fibrosis) (Fig. 1B). MRIs were obtained due to ongoing pediatric research in MRI of fatty liver and lack of readily available Fibroscan access.

Given her persistently elevated ALT of 74 U/L and positive smooth muscle antibody titer of >1:20, a repeat liver biopsy was obtained 2 years after presentation to rule out autoimmune hepatitis. It showed steatohepatitis with extensive bridging fibrosis and nodularity consistent with cirrhosis (stage 4) (Fig. 2B). Over 20 months, the patient’s liver fibrosis had progressed from stage 1 to 4. Repeat MRI and elastography 27 months after presentation showed worsened fat fraction of 35.1% and liver stiffness of 4.5–5.6 kPa (>5 kPa correlates to stage 4 fibrosis or cirrhosis).

Despite compliance with weekly appointments in a multidisciplinary weight loss clinic, lifestyle modifications, and metformin, the patient had persistently elevated BMI percentile at the 98th percentile, elevated ALT of 129 U/L, and worsened steatohepatitis on imaging. As she had not lost a significant amount of weight, metformin was stopped, and she was started on subcutaneous liraglutide 1.8 mg daily by endocrinology 3 years after initial presentation to help augment weight loss. She was titrated to 2.4 mg and then 3 mg daily over 4 months before decreasing to 1.8 mg daily due to insurance.

Two years after starting liraglutide and 5 years after her initial presentation, her BMI percentile remained stable at the 97th percentile. However, her ALT improved to 28 U/L. This occurred although she had limited physical activity and clinic follow up while taking liraglutide. Repeat MRI and elastography 65 months after presentation showed improved liver fat fraction of 13.8% and liver stiffness of 2.2–3.3 kPa (2.9–3.5 kPa correlates to stage 1–2 fibrosis).

## DISCUSSION

The pathogenesis of NAFLD is not fully understood, but it is thought that liver fat accumulation leads to lipotoxicity, causing inflammation, apoptosis, and reactive oxygen species release. This leads to a cycle of injury and healing, ultimately resulting in fibrosis (2). Insulin resistance, commonly seen in patients with NAFLD (3), may play a role by upregulating liver lipogenesis. This is exacerbated by insulin signaling pathway defects caused by fatty acid accumulation in hepatocytes (2).

Liraglutide stimulates insulin synthesis and secretion by pancreatic islet cells. It is used to improve glycemic control and decrease the risk of cardiovascular events in patients with type 2 diabetes mellitus. Additionally, it is thought to slow gastric emptying and increase satiety via the brain appetite centers, thus decreasing calorie and fat intake.

Though our patient did not lose a significant amount of weight after starting liraglutide, her ALT decreased from 120 to 28 U/L. She had a decreased liver fat fraction from 35.1% to 13.8%, and her liver stiffness improved from 4.49–5.61 kPa to 2.2–3.3 kPa, reflecting improvement from stage 4 fibrosis or cirrhosis to normal or stage 1–2 fibrosis. Liraglutide has previously been seen to improve NASH with and without fibrosis in pediatric patients (4), however it has not been reported in NASH-related cirrhosis.

Of note, the patient was not taking liraglutide daily, but only 3 times weekly. This was due to the subcutaneous administration and a perceived adverse smell. Additionally, the dose was titrated up to 3 mg daily, which is the Food and Drug Administration–approved dose for weight loss. She took this dose for 4 months before decreasing to 1.8 mg daily, which is the treatment dose for type 2 diabetes mellitus. With this decreased dose of liraglutide, she was still seen to have improvement of her NASH-related cirrhosis without significant improvement in her BMI percentile, suggesting that even the lower dose of liraglutide has hepatic effects independent of effects related to weight loss.

Our patient was not compliant with lifestyle modifications, which unfortunately is not atypical with the challenges in treating obesity. The improvement in our patient’s NASH-related cirrhosis with the use of liraglutide offers a therapeutic option when faced with the challenges in adherence to lifestyle interventions. The pubertal timing of our patient concurrent with the improvement of the metabolic profile is a potential confounder, as the surge of growth hormone during the onset of puberty may cause metabolic improvements. However, the persistence of the improvement beyond 14–15 years of age, which is the expected adolescent growth hormone peak effect, decreases the effects of this confounder and encouragingly suggests liraglutide as more likely to explain the metabolic improvements. While there are anecdotal reports of patients with advanced cirrhosis improving without intervention over time due to the poorly understood mechanisms of NASH progression, patients’ improvement with liraglutide is encouraging and future larger studies may confirm this finding.

This case shows the importance of considering the use of glucagon-like peptide-1 receptor agonists in patients for whom lifestyle interventions have not been adequate to improve NAFLD. The difficulties in patient compliance compel further investigation into other options (eg, weekly or oral semaglutide, which recently was shown to decrease BMI more than lifestyle interventions alone (5)). By stopping and potentially reversing the progression of NAFLD, these patients can avoid the many associated morbidities.

## ACKNOWLEDGMENTS

The parents of the child referenced in the case report provided informed consent for publication of the details of this case.
